# Transcriptome Profiling Predicts New Genes to Promote Maize Callus Formation and Transformation

**DOI:** 10.3389/fpls.2019.01633

**Published:** 2019-12-20

**Authors:** Xuemei Du, Ting Fang, Yan Liu, Liying Huang, Maosen Zang, Guoying Wang, Yunjun Liu, Junjie Fu

**Affiliations:** ^1^ Institute of Crop Sciences, Chinese Academy of Agricultural Sciences, Beijing, China; ^2^ College of Agronomy and Biotechnology, China Agricultural University, Beijing, China

**Keywords:** maize, callus induction, RNA-Seq, AP2 transcription factors, *Baby Boom*

## Abstract

Maize transformation is highly based on the formation of embryonic callus, which is mainly derived from scutellum cells of the immature maize embryo. However, only a few genes involved in callus induction have been identified in maize. To reveal the potential genes involved in the callus induction of maize, we carried out a high-throughput RNA sequencing on embryos that were cultured for 0, 1, 2, 4, 6, and 8 days, respectively, on a medium containing or lacking 2,4-dichlorophenoxyacetic acid. In total, 7,525 genes were found to be induced by 2,4-dichlorophenoxyacetic acid and categorized into eight clusters, with clusters 2 and 3 showing an increasing trend related to signal transmission, signal transduction, iron ion binding, and heme binding. Among the induced genes, 659 transcription factors belong to 51 families. An AP2 transcription factors, *ZmBBM2*, was dramatically and rapidly induced by auxin and further characterization showed that overexpression of *ZmBBM2* can promote callus induction and proliferation in three inbred maize lines. Therefore, our comprehensive analyses provide some insight into the early molecular regulations during callus induction and are useful for further identification of the regulators governing callus formation.

## Introduction

Tissue culture technology has been widely used in breeding programs, genetic engineering, and fundamental studies (Yadava, 2017). *Agrobacterium*-mediated transformation and particle bombardment are widely used tissue culture technology in cereals, both of which require callus induction and selection (Shrawat and Lörz, 2006). Sugimoto et al. (2010) suggested that callus are induced through lateral root initiation pathways in *Arabidopsis thaliana*. The exogenous application of auxin and cytokinin is necessary for *in vitro* callus induction for numerous plant species (Ikeuchi et al., 2013). Auxin signaling is transduced by auxin response factors (ARF), especially ARF7 and ARF19, which can activate the expression of the LATERAL ORGAN BOUNDARIES DOMAIN (LBD) and E2F TRANSCRIPTION FACTOR a (E2Fa) transcription factors (Fan et al., 2012; Ikeuchi et al., 2013). In *A. thaliana*, overexpression of *LBD16*, *LBD17*, *LBD18*, and *LBD29* were enough to induce callus with a similar appearance to the callus induced on callus-inducing-medium (Fan et al., 2012). With the exception of the ARF transcription factor, WUSCHEL-related homeobox 11 (*WOX11*) and JUMONJI C DOMAIN-CONTAINING PROTEIN 30 (*JMJ30*) can also regulate the LBD genes in *A. thaliana* (Liu et al., 2014; Lee et al., 2018). It was reported that LBD proteins interact with the basic leucine zipper (bZIP) transcription factor to promote callus formation (Xu et al., 2018). Furthermore, auxin downregulates the KIP-RELATED PROTEIN (KRP) genes encoding cyclin-dependent kinase (CDK) inhibitors, which have been identified as regulator inhibitors in callus formation (Anzola et al., 2010).

Callus formation can be induced by cytokinin and wounds. The critical components involved in this process include type-B ARRs and the ethylene response factor (ERF) subfamily of AP2 (AP2/ERF) transcription factors (Ikeuchi et al., 2019). Overexpression of *ARR1* can induce callus formation (Sakai, 2001). Interestingly, overexpression of *ARR1* or *ARR21* lacking the phosphorylation domain can induce callus formation without exogenous plant hormones (Sakai, 2001; Tajima et al., 2004). The AP2/ERF transcription factor WOUND INDUCED DEDIFFERENTIATION1 (*WIND1*) is a central regulator of wound-induced cellular reprogramming by upregulating the expression of the ENHANCER OF SHOOT REGENERATION1 (*ESR1*) gene, which encodes another AP2/ERF transcription factor in *A. thaliana* (Iwase et al., 2017).

Callus formation can also be influenced by embryonic or meristematic fate-related genes. Overexpression of master regulators in embryonic fate, such as LEAFY COTYLEDON 1 (*LEC1*), LEAFY COTYLEDON 2 (*LEC2*), BABY BOOM (*BBM*), and meristem fate WUSCHEL (*WUS*) could induce callus formation (Wójcikowska et al., 2013; Yang et al., 2014; Zheng et al., 2016; Ikeuchi et al., 2019). In particular, *BBM*, an AP2/ERF transcription factor, can ectopically induce somatic embryogenesis in many plant species, such as *A. thaliana*, *Brassica napus*, *Theobroma cacao*, and *Zea mays* (Boutilier, 2002; Florez et al., 2015; Lowe et al., 2016). *BBM* is a member of the AINTEGUMENTA-LIKE (AIL) family of AP2/ERF domain transcription factors, which are expressed in all dividing tissues and have central roles in developmental processes (Horstman et al., 2014). In *A. thaliana*, AIL family contains eight genes, with single AIL knockout mutants showing hardly any defects. However, double or triple mutants have stronger phenotypes in different developmental processes (Krizek, 2015). The overexpression of AIL proteins can induce somatic embryogenesis and ectopic organ formation (Boutilier, 2002; Tsuwamoto et al., 2010). In *A. thaliana*, *BBM* was reported to induce somatic embryogenesis *via* regulating *LEC1*, *LEC2*, *FUSCA3* (*FUS3*), and ABSCISIC ACID INSENSITIVE3 (*ABI3*) (Horstman et al., 2017).

Maize provides an important source of food, feed, and industrial raw materials worldwide and is one of the prime targets for genetic manipulation (Que et al., 2014). Genetic transformation in maize usually requires the formation of embryonic callus, which is mainly derived from the scutellum cells of immature maize embryos (Ishida et al., 2007). However, embryonic callus is difficult to induce in many maize lines, restricting the scope of maize transformation (Ma et al., 2018). To cope with this question, transformation method without callus growth has been developed using *ZmBBM* and *Wus2* (Lowe et al., 2018). A previous study suggested that five quantitative trait loci can explain 82% of the phenotypic variance for embryogenic callus formation in an A188 × B73 population (Armstrong et al., 1992). Immature embryo from the maize line A188 at 0, 24, 36, 48, and 72 hours after induction were analyzed by high-throughput RNA sequencing (RNA-Seq), and the results showed that the expression of the genes involved in stress response and hormone transport was increased (Salvo et al., 2014). A multi-omic data analysis on different stages of callus formation of maize line 18-599R revealed that several genes and miRNAs related to metabolism, cellular processes, and signaling may function in callus induction (Shen et al., 2012; Shen et al., 2013).

Until now, few genes related to callus induction have been identified in maize except for *ZmBBM* and *Wus2* (Lowe et al., 2016). Because callus induction is influenced by genotype, it has been speculated that novel genes could be identified using different maize lines. In the present study, we carried out RNA sequencing of maize line CAL to identify crucial genes involved in maize callus formation, including *ZmBBM2*. Furthermore, we confirmed that overexpression of *ZmBBM2* could promote the transformation efficiency of immature maize embryos in three inbred lines. Our research will help further identification of the crucial genes in callus induction.

## Materials and Methods

### Samples, RNA Isolation, and Sequencing

Plants from the inbred maize line CAL were grown in a greenhouse with a 16 h/8 h light/dark cycle at 20–25°C. Immature embryos were collected from the maize ears 10–12 days after pollination. The immature embryos (1.0–1.2 mm) were isolated and placed on N6 medium with 1.5 mg/L 2,4-dichlorophenoxyacetic acid (2,4-D) and subjected to aphotic culturing at 27°C. After culturing for 0, 1, 2, 4, 6, and 8 days (D0, D1, D2, D4, D6, and D8) at 27°C, the immature embryos were collected and stored at −70°C for RNA extraction. Meanwhile, immature embryos cultured without 2,4-D (N1, N2, N4, N6, and N8) were also collected as control. More than 50 immature embryos were collected for each sample, and three biological replicates of each sample were used for the following RNA-Seq.

The extracted RNA was inserted into a 1% agarose gel to assess the RNA integrity. RNA yield and purity were checked using a Nano-drop ND-1000. mRNAs were isolated from total RNA using oligo(dT) magnetic beads (Illumina, San Diego, CA, USA). RNA fragmentation, cDNA synthesis, and PCR amplification were performed according to the Illumina RNA-Seq protocol. cDNA libraries were sequenced with a read length of 150 bp (paired-end) using an Illumina HiSeq 2500 System at Berry Genomics (Beijing, China).

### RNA-Seq Reads Mapping and Expression Analysis

All clean reads with high quality from each sample were mapped to the maize B73 AGPv3.27 reference genome using Tophat2 version 2.1.0 with the strict parameters “-i 5 -I 60000 –mate-inner-dist 238 –mate-std-dev 45”, which allow a default of two mismatches (Kim et al., 2013). Next, SAMtools v1.2 was used to filter the reads with a mapping quality score under 50 (Li et al., 2009). The filtered reads were used as inputs into the *DESeq2* R package and fragments per kilobase of transcript per million reads mapped values were calculated to evaluate the expression levels of the genes. Differentially expressed genes (DEGs) were identified with the criteria of |log2fold change| ≥1 and false discovery rate (FDR) adjusted p-value < 0.01. All the upregulated and downregulated DEGs at the same day with or without 2,4-D induction were compared and only the DEGs in the 2,4-D induced samples were identified as the induced genes.

### Cluster Analysis and Gene Ontology Analysis

The *Mfuzz* R package was used to cluster the expression levels of all induced genes, which is based on the soft clustering of time series gene expression data, called c-means clustering (Lokesh and Matthias, 2007). An optimal setting of fuzzifier m, which prevented clustering of random gene expression, was estimated as 1.722. To characterize the functional categories among the induced genes, we used the agriGO online website (http://systemsbiology.cau.edu.cn/agriGOv2/) to perform gene ontology analysis (Tian et al., 2017). The hypergeometric statistical test method followed by FDR correction (FDR < 0.05) was used to enrich significant functional terms.

### *Agrobacterium*-Mediated Transformation of Immature Embryos

The coding sequence of *ZmBBM2* were amplified from the cDNA of immature embryos at D8, and then inserted into the binary vector pCambia3301 at the *Nco*I and *Bst*EII restriction sites, to produce pCambia3301-35S-*ZmBBM2* expression vector. This vector was transformed into *Agrobacterium* strain EHA105. Immature embryos from maize inbred lines CAL, Zong31, and B73 were transformed according to the *Agrobacterium*-mediated method (Chen et al., 2018). The tissues of Zong31 and B73 were cultured and selected under dim light (10 μmol m^−2^ s^−1^). The tissues of CAL were cultured and selected under both darkness and dim light (10 μmol m^−2^ s^−1^), respectively. Resistant calli were placed in Murashige and Skoog medium for regeneration under fluorescent white light in a 16/8 h light/dark cycle. Genomic DNA was extracted from the calli using the CTAB method and the presence of transgene was confirmed by PCR amplification using the primers listed in **Table S1**. The calli with amplified fragments were confirmed as positive. The transformation efficiency was calculated as by the number of the positive calli per the infected embryos. The transformation experiment was performed with three independent replicates.

### Analysis of the Effect of *ZmBBM2* Overexpression on Tissue Growth

The diameter of CAL positive calli transformed with pCambia3301 or pCambia3301-35S-*ZmBBM2* were measured, respectively, after three rounds of 2-week long selection with bialaphos. The data came from three biological replicates, and four calli were measured in each biological replicate. The positive calli were also collected for RNA extraction and quantitative real-time PCR analysis (qRT-PCR).

### Quantitative Real-Time PCR

The first-strand cDNA synthesis was performed with the M-MuLV reverse transcriptase (Promega) using total RNA as the template. qRT-PCR was carried out with three biological replicates of each sample using the ABI 7300 system. The relative transcriptional levels were calculated using the 2^-ΔΔ^ *^C^* ^t^ method or 2^-Δ^ *^C^* ^t^ method (Livak and Schmittgen, 2001) with actin as a housekeeping gene. The primers used in qRT-PCR analysis are listed in **Table S1**.

## Results

### Whole Transcriptome Profiling

RNA-seq experiments were performed to investigate global transcriptome changes during callus induction in the maize inbred line CAL, using immature embryos cultured on a medium with 2,4-D for 0, 1, 2, 4, 6, and 8 days. To exclude the influence of the culture medium components, RNA was also extracted from immature embryos cultured on a medium without 2,4-D and sequenced. Approximately 807 million (806,937,547) clean reads were mapped to the maize B73 reference genome (AGPv3.27, **Table S2**). The proportion of concordant pair alignment of all samples were more than 64.25% (64.25%~71.15%), which were then used for further analyses (**Table S2**).

**Figure 1:**
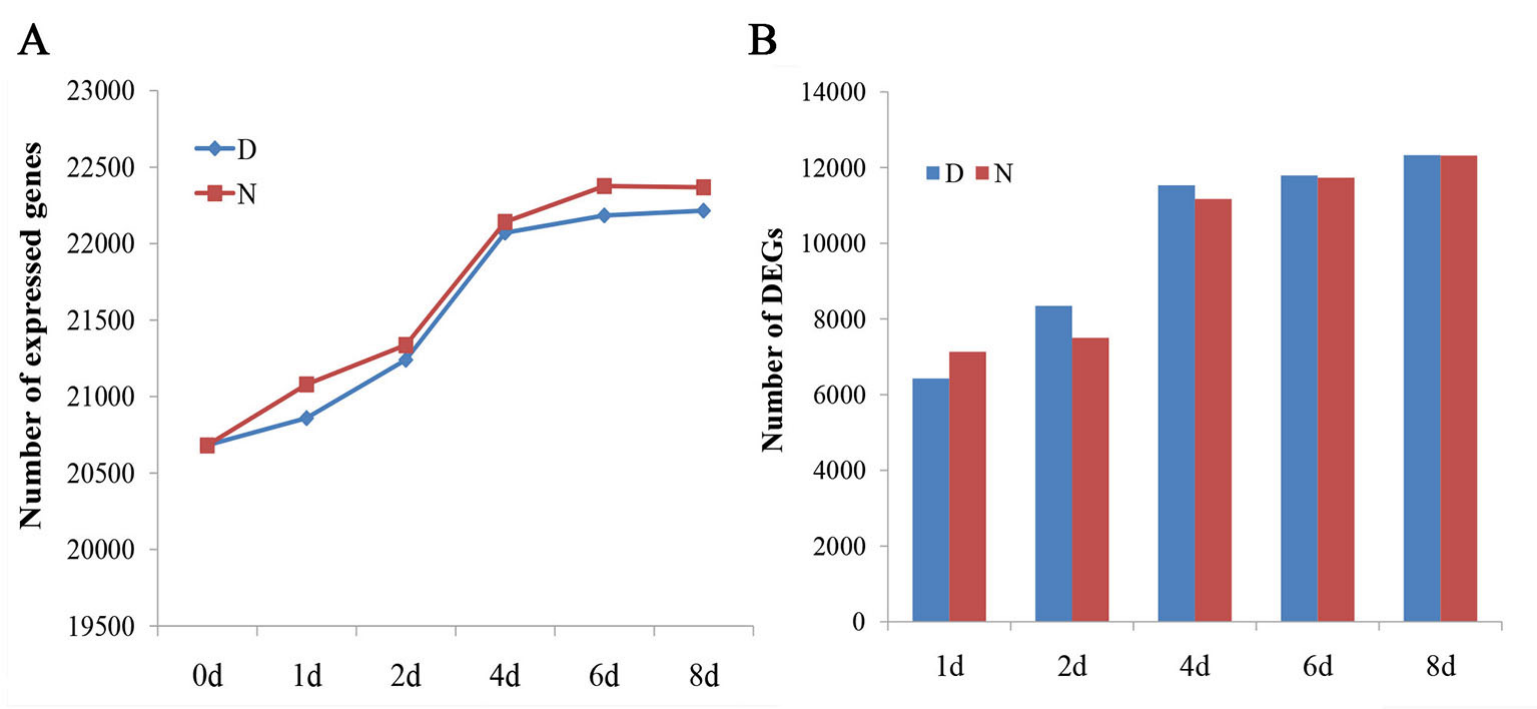
Analysis of global gene expression among different samples. **(A)** The number of expressed genes in different samples (fragments per kilobase of transcript per million reads mapped > 1). **(B)** The number of differentially expressed genes in different samples compared with D0. D and N indicate the embryos cultured on N6 medium with or without 1.5 mg/l 2,4-D, respectively.

To ensure high confidence in the genes expressed, the genes were analyzed only if their fragments per kilobase of transcript per million reads mapped values were >1 in at least one sample. For every sample, approximately 21,602 (20,681 to 22,375) genes were detected (**Figure 1A**). The DEGs were obtained by comparing the samples to D0 (|log2fold change| ≥1 and FDR < 0.01). The number of DEGs were all increased with and without the 2,4-D induction (**Figure 1B**).

### Induced Genes in Callus Formation

**Figure 2:**
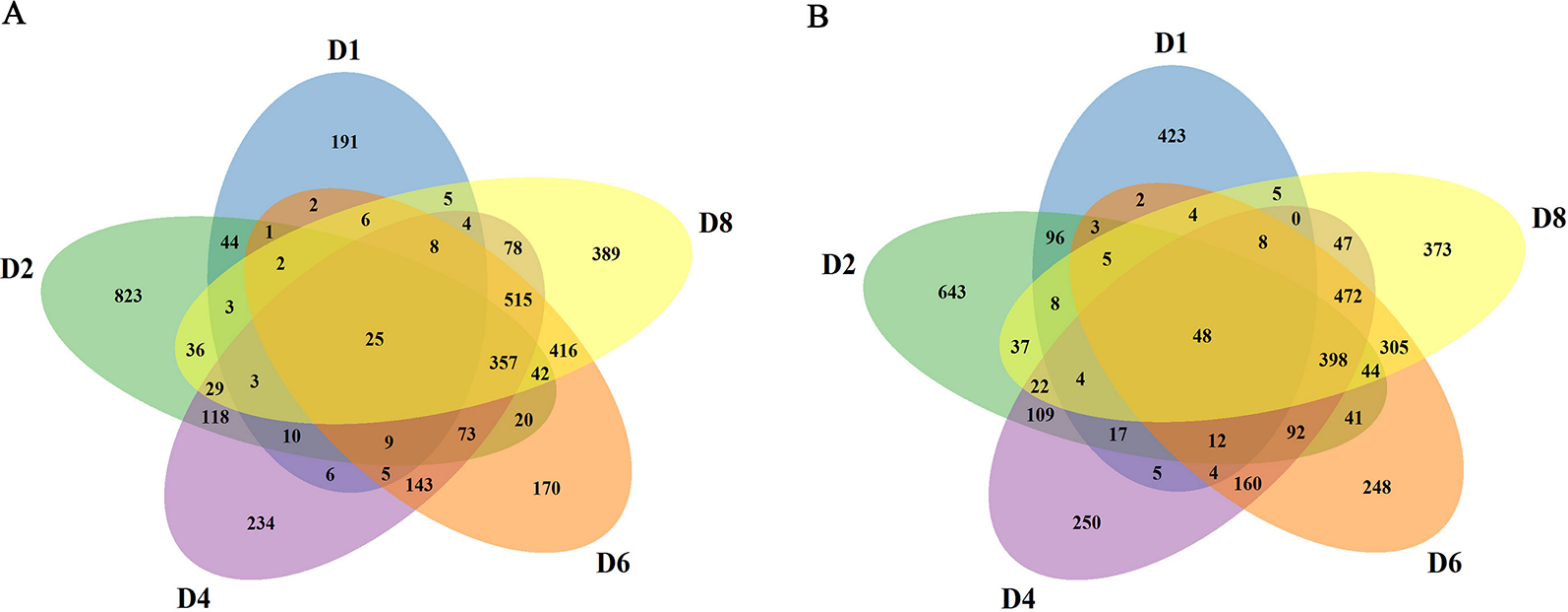
Summary of the induced genes. **(A)** Comparison of upregulated genes at different stages. **(B)** Comparison of downregulated genes at different stages.

In order to obtain the induced genes during callus formation, the upregulated and downregulated DEGs at the same day with or without 2,4-D induction were compared. Only the DEGs in the 2,4-D induced samples were identified as the induced genes. In total, 7,525 induced genes were obtained in callus induction (**Table S3**). Among them, 324, 1,595, 1,617, 1,794, and 1,918 genes were identified as the upregulated genes for D1, D2, D4, D6, and D8, respectively (**Figure S1A**). A total of 644, 1,579, 1648, 1,846, and 1,780 genes were downregulated for D1, D2, D4, D6, and D8, respectively (**Figure S1B**). Only 25 upregulated and 48 downregulated genes overlapped between different days of induction, indicating a notable variation of expression level changes during callus formation (**Figures 2A**, **B**). qRT-PCR analysis were performed on 10 genes to verify the RNA-seq data, and the expression changes of these genes revealed by qRT-PCR were similar to those observed in RNA-seq data, indicating the accuracy of RNA-seq data (**Figure S2**).

### Characterization of Induced Genes Grouped by Cluster Analysis

**Figure 3:**
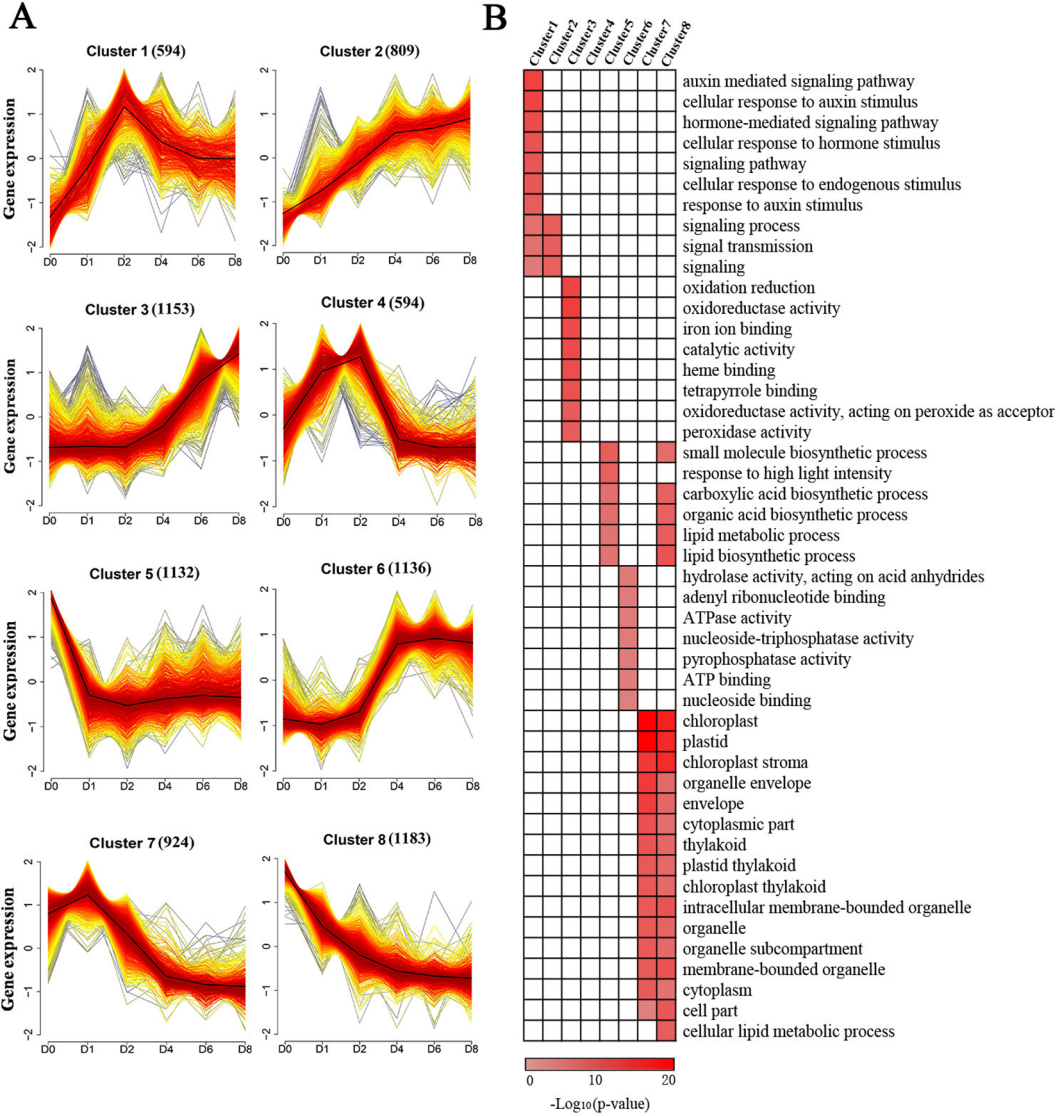
Cluster analysis and gene ontology functional categorization of the induced genes. **(A)** Clustering of the induced genes based on their expression patterns at D0, D1, D2, D4, D6, and D8. The numbers in parentheses indicate the number of genes in a cluster. The x-axis represents the sample and the y-axis represents centralized and normalized expression values. The black lines are the mean expression trends of the induced genes within each cluster. **(B)** Functional categories within the different clusters. Only significant categories (false discovery rate < 7.90E-4) are displayed.

To provide further insights into the functional transitions in callus induction, 7,525 induced genes were grouped to eight clusters according to their expression patterns using the Mfuzz R program, which was followed by a gene ontology annotation to assign functional categories for each gene cluster (**Table S4**). Clusters 2 and 3 show an increasing trend with induction (**Figure 3A**). Induced genes in cluster 2, such as *ARF20* and *ARF27*, were upregulated between D1 and D4, and they are mainly related to external stimuli, signaling transmissions, signaling transductions, intracellular signaling cascades (**Figure 3B**). The cluster 3 genes, such as *LBD24*, were rapidly induced after D4, and they are related to oxidation-reduction reactions, iron ion binding, heme binding, peroxidase activity, and transferase activity (**Figures 3A**, **B**).

Clusters 1 and 4 exhibited similar expression patterns showing peak expressions at D2 and dropping thereafter (**Figure 3A**). These two clusters included genes related to hormone-mediated signaling pathways and cellular response to these stimuli, especially in relation to the presence of auxin, such as *IAA*, *PIN*, and *ZmWOX11* (**Figure 3B**). The genes in cluster 6 exhibited rapid upregulation at D4, followed by maintaining a high level of expression (**Figure 3A**). These genes, such as *ZmBBM*, were primarily related to hydrolase activity and nucleoside binding (**Figure 3B**). The genes in clusters 5, 7, and 8 show a decreasing trend with 2,4-D induction (**Figure 3A**). The genes in these clusters were related to small molecule biosynthetic processes, plastids, envelopes, and intracellular membrane-bounded organelles (**Figure 3B**).

### Identification of the Induced Transcription Factors During Callus Induction

**Figure 4:**
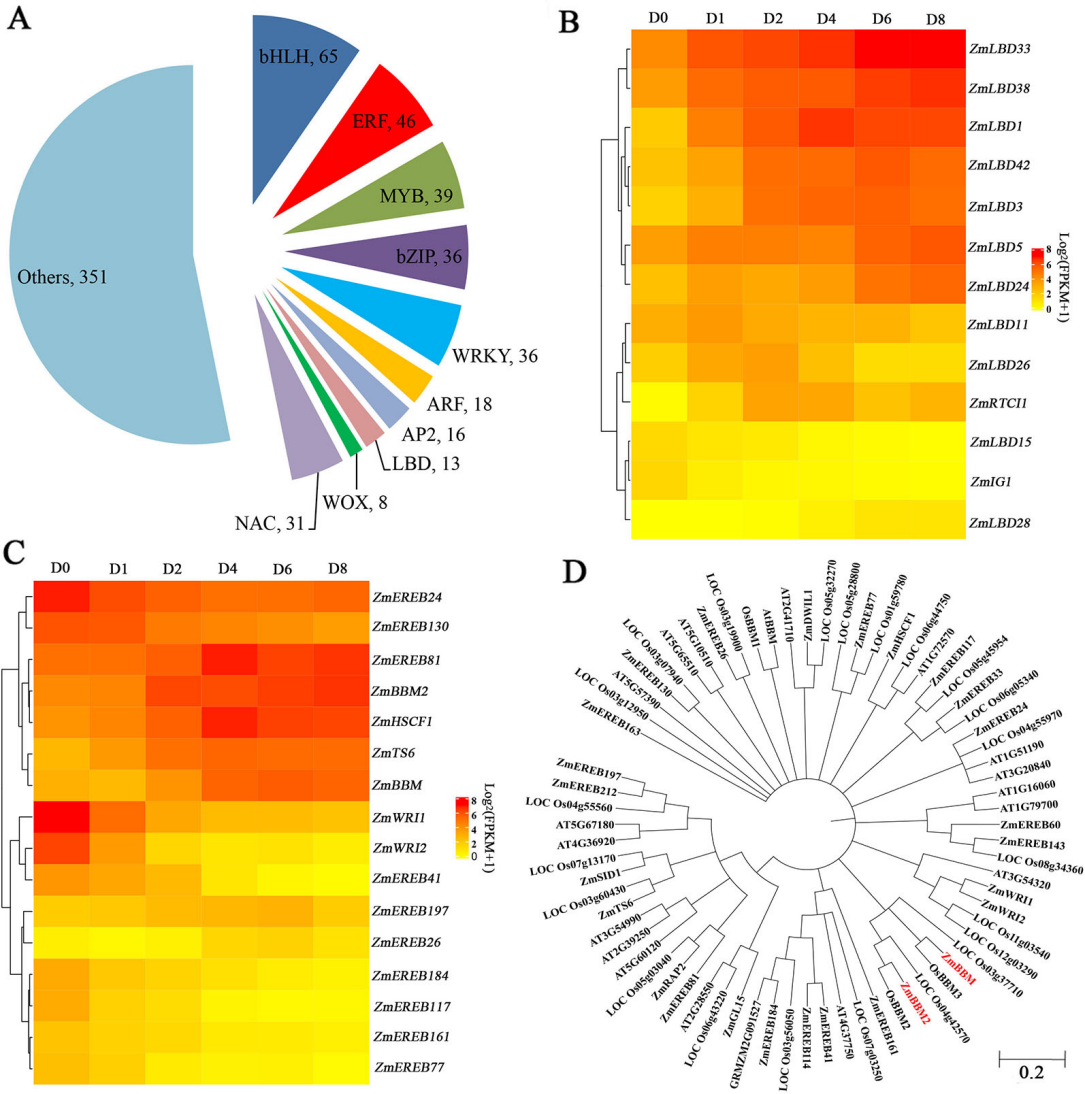
Transcription factors analysis. **(A)** Pie chart showing the TF number and families. **(B)** Heatmap of LBD TFs with altered expression levels after induction. The color scale of yellow (low) and red (high) represents the normalized expression levels. **(C)** Heatmap of AP2 TFs with altered expression levels after induction. The color scale of yellow (low) and red (high) represents the normalized expression levels. **(D)** Phylogenetic trees of the AP2 TFs in *Arabidopsis thaliana*, *Oryza sativa*, and *Zea mays*.

Among the induced genes, 659 transcription factors were found, belonging to 51 gene families (**Figure 4A**). Among these genes, 65 genes are BASIC/HELIX–LOOP–HELIX (bHLH) transcription factors, followed by ERF transcription factors with 46 members that showed expression changes after induction (**Figure 4A**). Other transcription factors include 39 MYBs, 36 bZIPs, 36 WRKYs, 31 NACs, and 18 ARFs that exhibited obvious expression changes after induction (**Figure 4A**). Thirteen LBD transcription factors, which play roles in callus initiation and plant regeneration, showed upregulation at least in one time period, with the exception of *ZmIG1* and *LBD15* (**Figure 4B**, **Table S5**). In particular, *LBD5*, *LBD24*, *LBD33*, and *LBD38* showed continuous upregulation.

Among the 16 induced AP2 transcription factors, 9 genes belonging to clusters 5 and 8 were downregulated after induction, and other seven genes showed gene upregulation (**Figure 4C**, **Table S6**). *ZmTS6*, *ZmHSCF1*, and *ZmEREB81* exhibited peak expressions at D4. *ZmBBM*, *ZmEREB26*, and *ZmEREB197* exhibited peak expressions at D6. In comparison, *ZmBBM2*, were dramatically and rapidly induced from D2 (**Figure 4C**, **Table S6**). The amino acid sequence of *ZmBBM2* has a 78% similarity with *OsBBM2* and 43% similarity with *ZmBBM* (**Figure 4D**, **Figure S3**). According to MaizeGDB data, *ZmBBM2* is expressed only in the immature embryos and roots.

### Overexpression of *ZmBBM2* Promotes Callus Formation and Proliferation

**Figure 5:**
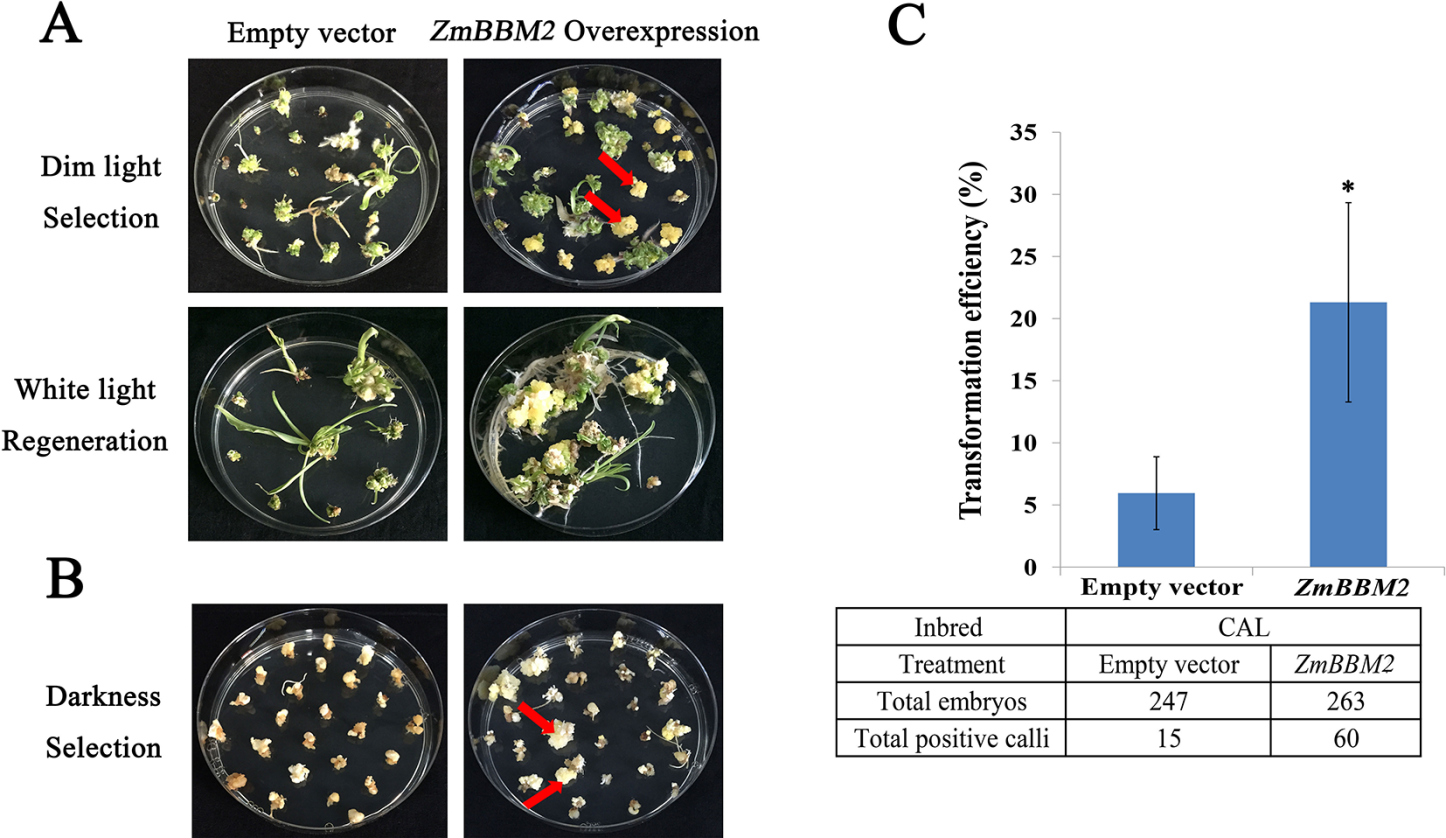
Overexpression of *ZmBBM2* promotes callus formation and proliferation in the CAL line. **(A)** Comparison of the control callus with the *ZmBBM2* overexpressed callus under dim light selection and callus regeneration under fluorescent white light (Arrow refers to the *ZmBBM2* positive callus). **(B)** Comparison of the control callus with the *ZmBBM2* overexpressed callus under darkness (Arrow refers to the *ZmBBM2* positive callus). **(C)** The comparison of transformation efficiency between *ZmBBM2* overexpression and the control in CAL line. Data was shown as average ± S.D. of three independent experiments. Experimental data was tested by student *t*-test analysis and asterisks in the column mean significant difference at *P* < 0.05 level. The numbers of total infected embryos and positive calli are shown under the histogram.

**Figure 6:**
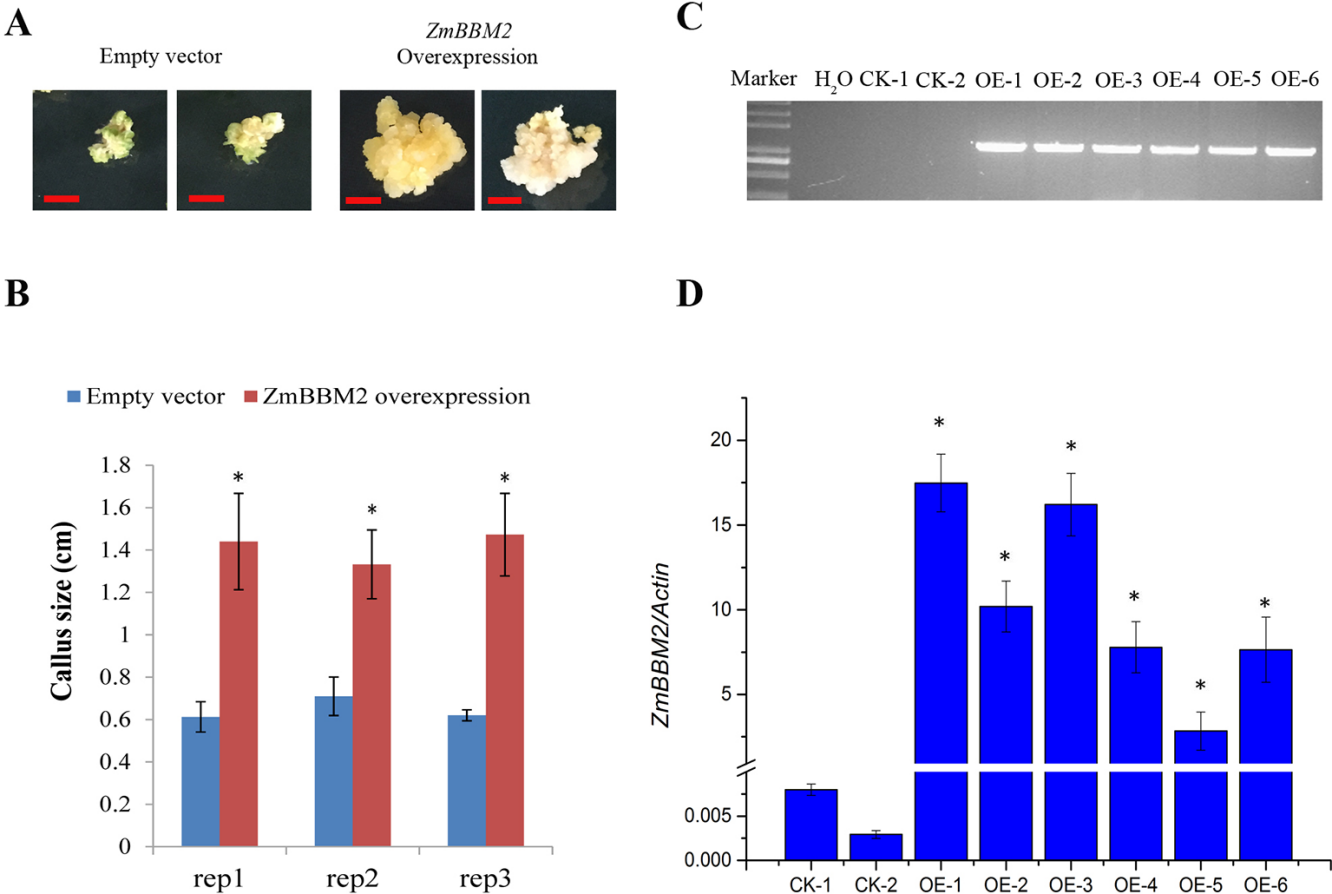
*ZmBBM2* overexpression increased callus size. **(A)** The representative positive calli for empty vector and *ZmBBM2* vector after three rounds of 2-week selection on the medium containing bialaphos. The left are two representative positive calli for empty vector, and the right are two representative positive *ZmBBM2* calli. Scale bar = 0.5 cm. **(B)** Callus size of the positive *ZmBBM2* calli and control calli. After three rounds of 2-week selection on the medium containing bialaphos, the callus size was determined based on the diameter of callus masses. The data of three independent experiment replications (rep1, rep2, and rep3) was presented. Error bars are the standard deviation of four independent calli. Asterisks in each column mean significant difference between *ZmBBM2* positive calli and control at *P* < 0.05 level. **(C)** PCR amplification of *ZmBBM2* in positive calli. M, DNA marker; CK-1 and CK-2, callus transformed with empty vector; OE1~OE6, *ZmBBM2* positive calli. These calli were selected randomly from other positive calli, which were different from those shown in A and B. **(D)** *ZmBBM2* expression level in the *ZmBBM2* positive and control calli shown in C; Data was shown as average ± S.D. of three independent experiments. Experimental data was tested by student *t*-test analysis and asterisks in each column mean significant difference between *ZmBBM2* positive calli and control at *P* < 0.05 level.

**Figure 7:**
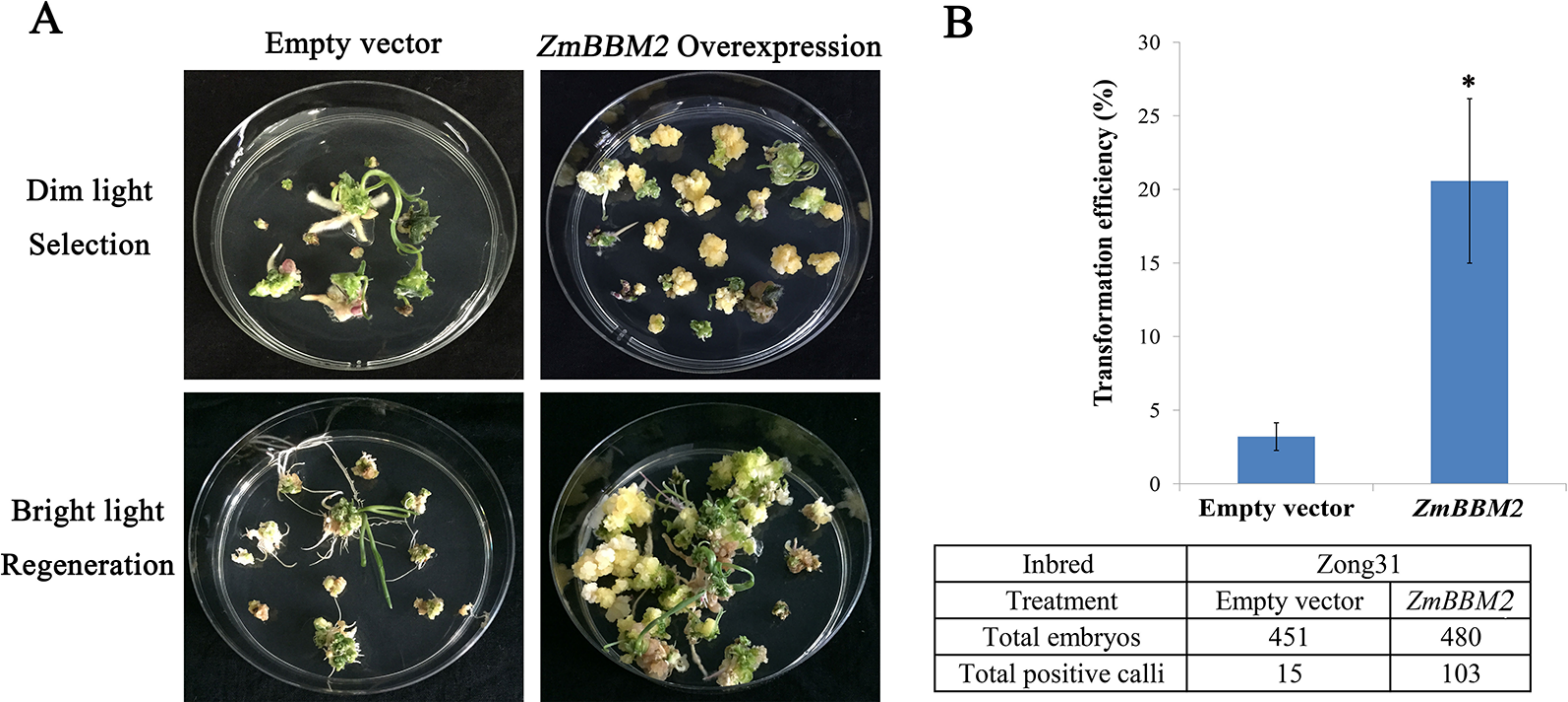
Overexpression of *ZmBBM2* promotes callus formation and proliferation in the Zong31 line. **(A)** Comparison of the control callus with the *ZmBBM2* overexpressed callus under dim light selection and callus regeneration under fluorescent white light (Arrow refers to the *ZmBBM2* positive callus); **(B)** The comparison of transformation efficiency between *ZmBBM2* overexpression and the control in the Zong31 line. Data was shown as average ± S.D. of three independent experiments. Experimental data was tested by student *t*-test analysis and asterisks in the column mean significant difference at *P* < 0.05 level. The numbers of total infected embryos and positive calli are shown under the histogram.

To verify the role of *ZmBBM2* in callus formation, *ZmBBM2* was amplified and ligated into the plasmid pCambia3301, under the control of the cauliflower mosaic virus 35S promoter. The plasmid was then transformed into immature maize embryos of CAL and Zong31, both of which have high transformation efficiency and are frequently used in maize transformation. The transformation efficiency of *ZmBBM2* overexpression in CAL and Zong31 were significantly increased compared with that in the controls (6% in the control to 21% with overexpression in CAL, and from 3% in the control to 20% with overexpression in Zong31) (**Figures 5C** and **7B**). Compared with control samples, the *ZmBBM2* positive calli were bigger and exhibited rapid growth in darkness and dim light (**Figures 5A, B**, **6A**, **7A**). After three rounds of 2-week selection on the medium containing bialaphos (**Figure S4**), the callus size was determined based on the diameter of callus masses, and the sizes of *ZmBBM2* positive calli were higher than the control calli (**Figure 6B**). The positive calli was confirmed by PCR amplification of the fragment containing *ZmBBM2* and *NOS* terminator using the specific primers, and the results showed that specific fragments could only be amplified from *ZmBBM2* positive calli (**Figure 6C**, **Table S1**). Significantly higher expression levels of *ZmBBM2* in these positive calli were detected compared to the controls (**Figure 6D**).

The *ZmBBM2* construct was also transformed into immature embryos of inbred line B73 that is a recalcitrant line for maize transformation. In total, only four positive calli were obtained with empty vector transformation from 247 immature embryos. In comparison, six positive calli were obtained with overexpression of *ZmBBM2* from 136 immature embryos of the B73 line (**Figure S5**). Only one experiment was performed for the B73 line transformation, and this preliminary result indicate *ZmBBM2* can increase transformational efficiency in this recalcitrant maize line.

## Discussion

Improving transformation efficiency is necessary for the functional annotation of the maize genome and the generation of transgenic maize with breeding value (Altpeter et al., 2016). The efficiency of embryonic callus induction is an important factor influencing the efficiency of callus-dependent transformation in maize (Yadava et al., 2017). However, it is difficult for many inbred maize lines to undertake dedifferentiation and induce embryogenic callus (Ma et al., 2018). Understanding the molecular mechanism of maize embryogenic callus induction could facilitate maize transgene production. In this study, we conducted a high-throughput RNA sequencing of CAL, an inbred line with a high efficiency of embryonic callus induction, to identify elaborate transcript level changes during callus formation. We found 7,525 induced genes by comparing the DEGs in the samples with or without 2,4-D induction.

Previous studies had shown that callus could be induced from aerial organs and resembles the tip of a root meristem (Sugimoto et al., 2019). The root meristem regulator genes, such as *WOX5* and *WOX11*, had been verified to play important roles in callus initiation in *A. thaliana* and *Oryza sativa* (Hu et al., 2017; Cheng et al., 2018; Liu et al., 2018a; Sang et al., 2018). In this study, *ZmWOX11* showed peak expression at D2 and dropped afterward. In contrast, *ZmWOX5a* and *ZmWOX5b* exhibited upregulation after D2, implying an involvement in the first- and second-step cell fate transition in callus initiation like in *A. thaliana* and *O. sativa*. In addition, LBD transcription factors, which are downstream of *WOX11*, *ARF*, and *JMJ30*, act as the key molecules governing genes in callus formation (Liu et al., 2018b; Ikeuchi et al., 2019). In *A. thaliana*, overexpression of some LBD transcription factors is sufficient to induce callus with a similar appearance to CIM-induced callus (Fan et al., 2012). Moreover, *LBD16* forms a complex with the *bZIP59* transcription factor and directly activates downstream genes, such as *FAD-BD* (Xu et al., 2018). In this study, 13 LBD genes were identified in the induced genes and four of them increased dramatically at all the time points. In addition, the transcription factor *ZmbZIP65* showed downregulation after induction, similar to the homologous gene *bZIP59* in *A. thaliana*. These findings indicate that plant cells may have common mechanisms for callus induction at least in *A. thaliana*, *O. sativa*, and maize.

Callus induction is influenced by multiple plant hormones, particularly auxin and cytokinin (Ikeuchi et al., 2013). Although the callus induction medium used in the study only contained auxin, many genes involved in cytokinin, jasmonates (JAs), abscisic acid, ethylene, and gibberellin production also had obvious expression changes. In cytokinin signaling, ARRs are the primary regulatory genes in callus induction and an overexpression of *ARR1* in cytokinin-containing media can induce callus formation (To and Kieber, 2008). In the current study, five *ARR* genes exhibited expression changes, with *ZmARR1*, *ZmARR7*, and *ZmARR12* having sustained upregulation during callus induction. Xu (2018) recently reported that JAs serve as a wound signal during *de novo* root regeneration in *A. thaliana*. F-box protein CORONATINE INSENSITIVE1 (COI1) and JA ZIM domain (JAZ) are the main repressor proteins in JA signaling (Katsir et al., 2008). The bHLH transcription factor MYC2 is the core regulator for stimulating the transcription of stem cell regulators PLT1/2 (Chen et al., 2011). In our study, the orthologous genes of *COI1*, *JAZ*, and *MYC2* showed obvious expression changes with induction. In addition, JA can activate *ERF109* and the downstream gene *CYCD6;1*, which promotes regeneration (Zhang et al., 2019). In our study, 46 ERF genes were differentially expressed. Moreover, the expressions levels of several genes involved in ethylene, brassinosteroid, abscisic acid, and gibberellin signaling were changed during callus induction, suggesting that all these hormone signals may play roles in the callus formation.

The *BBM* gene belongs to the AIL family of the AP2/ERF domain transcription factor, overexpression of which can induce somatic embryogenesis and ectopic organ formation (Horstman et al., 2014). *BBM* was first reported in *Brassica campestris* and the overexpression of *BBM* could mediate somatic embryogenesis and enhanced plant regeneration in *Populus tomentosa*, maize, and other plant species (Jha and Kumar, 2018). *BBM* is mainly expressed in embryos and roots and regulates cell identity and growth with other AIL proteins (Horstman et al., 2014; Jha and Kumar, 2018). In *Glycine max*, *GmBBM1* was found to have a motif specific bbm-1 linking to the euANT2 motif identified in two other AIL proteins (El Ouakfaoui et al., 2010). The deletion of the euANT2 motif individually and with other motifs in *GmBBM1* prevented somatic embryos, implying functional differences between different AIL proteins (El Ouakfaoui et al., 2010). Lowe et al. (2016) showed that *ZmBBM* could improve the transformation efficiency of *Zea mays*, *Sorghum bicolor*, and *O. sativa*. In the current study, *ZmBBM2*, a new AIL protein, was found to have a 43% similarity to *ZmBBM*. However, *ZmBBM2* and *ZmBBM* showed different expression changes and belonged to different clusters. Further experiment in the study identified that the overexpression of *ZmBBM2* can promote callus formation and proliferation in different inbred lines. The transgenic seedlings ectopic expressing *ZmBBM* usually results in aberrant phenotypes (Lowe et al., 2016). We did not observe obvious aberrant phenotype for the mature transgenic plants overexpressing *ZmBBM2* (**Figure S6**), indicating that these two maize *BBM* genes may play roles with somewhat different mechanism. The promoter from maize phospholipid transferase protein gene have been used to drive *ZmBBM* gene to lessen the adverse impact of *ZmBBM* overexpression (Lowe et al., 2018). In this study we only used 35S promoter to drive *ZmBBM2*, and will check other weak or auxin-induced promoters, i.e. Nos promoter in the future experiments. Combining *ZmBBM* and *ZmWUS2* led to the direct formation of somatic embryos on the scutella, resulting in the callus-free transformation (Lowe et al., 2018). *ZmBBM2* in this study might also be used for the callus-free transformation, together with *ZmWUS2*. The results from our experiment can further an understanding of AIL proteins in maize and identify new genes for improving maize transformation efficiency.

## Conclusion

A high-throughput RNA sequencing were carried out on the embryos of maize line CAL and 7,525 genes were found to be induced by auxin. The induced genes were categorized to eight clusters and the cluster 2, 3 all showed an increasing trend with the induction. An AP2 transcription factors in cluster 2, *ZmBBM2*, were dramatically and rapidly induced by auxin, and further study showed that overexpression of *ZmBBM2* can promote callus induction and proliferation in three maize inbred lines. Therefore, our comprehensive analyses are useful for further identification of the regulators governing callus formation.

## Data Availability Statement

The sequence data generated in this study has been deposited in the NCBI and can be found using accession number PRJNA578052 (https://www.ncbi.nlm.nih.gov/bioproject/PRJNA578052).

## Author Contributions

GW, JF, and YJL designed the research. XD, TF, and YL performed the research. LH and MZ collected samples. XD, JF, and YJL wrote the article.

## Conflict of Interest

The authors declare that the research was conducted in the absence of any commercial or financial relationships that could be construed as a potential conflict of interest.

## Supplementary Material

Supplementary Figure S1 Summary of the induced genes among different samples. Comparison of upregulated and downregulated genes at (A) D1, (B) D2, (C) D4, (D) D6 and (E) D8.

Supplementary Figure S2 Correlations of the differential expression ratios between qRT-PCR and RNA-sequencing of ten induced genes among different samples.

Supplementary Figure S3 Multiple alignment of ZmBBM2 (GRMZM2G366434), OsBBM2 (LOC_Os02g40070) and ZmBBM (GRMZM2G141638) protein sequences. The identical residues are shaded in dark blue and conservative changes are shaded in light blue.

Supplementary Figure S4 Process of the positive callus selection. Immature maize embryos were infected with agrobacterium and co-cultured for three days, and then rested for seven days. The positive calli were selected on medium containing 3 mg/L bialaphos through three rounds of two-week selection. Arrows refer to the positive callus. scale bar=2 cm.

Supplementary Figure S5 Overexpression of ZmBBM2 promotes callus formation and proliferation in the B73. Arrow refers to the ZmBBM2 positive callus.

Supplementary Figure S6 The wide type and ZmBBM2 overexpression plants in the Zong31 line at 30 days after self-pollination. scale bar=20 cm.

Supplementary Table S1 The primers used in qRT-PCR, plasmid construction and genomic PCR amplification.

Supplementary Table S2 Statistics of RNA sequencing Reads of 2,4-D-treatment (D) and normal (N) maize immature embryos at different days.

Supplementary Table S3 Upregulated and downregulated induced genes at different days.

Supplementary Table S4 Clustering results of the 7525 induced genes.

Supplementary Table S5 The expression level change of the induced LBD transcription factors.

Supplementary Table S6 The expression level change of the induced AP2 transcription factors
