# Supplementary figures and images for: Transcriptome Profiling Predicts New Genes to Promote Maize Callus Formation and Transformation
Source: Front Plant Sci. 2019 Dec 20;10:1633. doi: 10.3389/fpls.2019.01633 (PMC6934073; doi:10.3389/fpls.2019.01633)

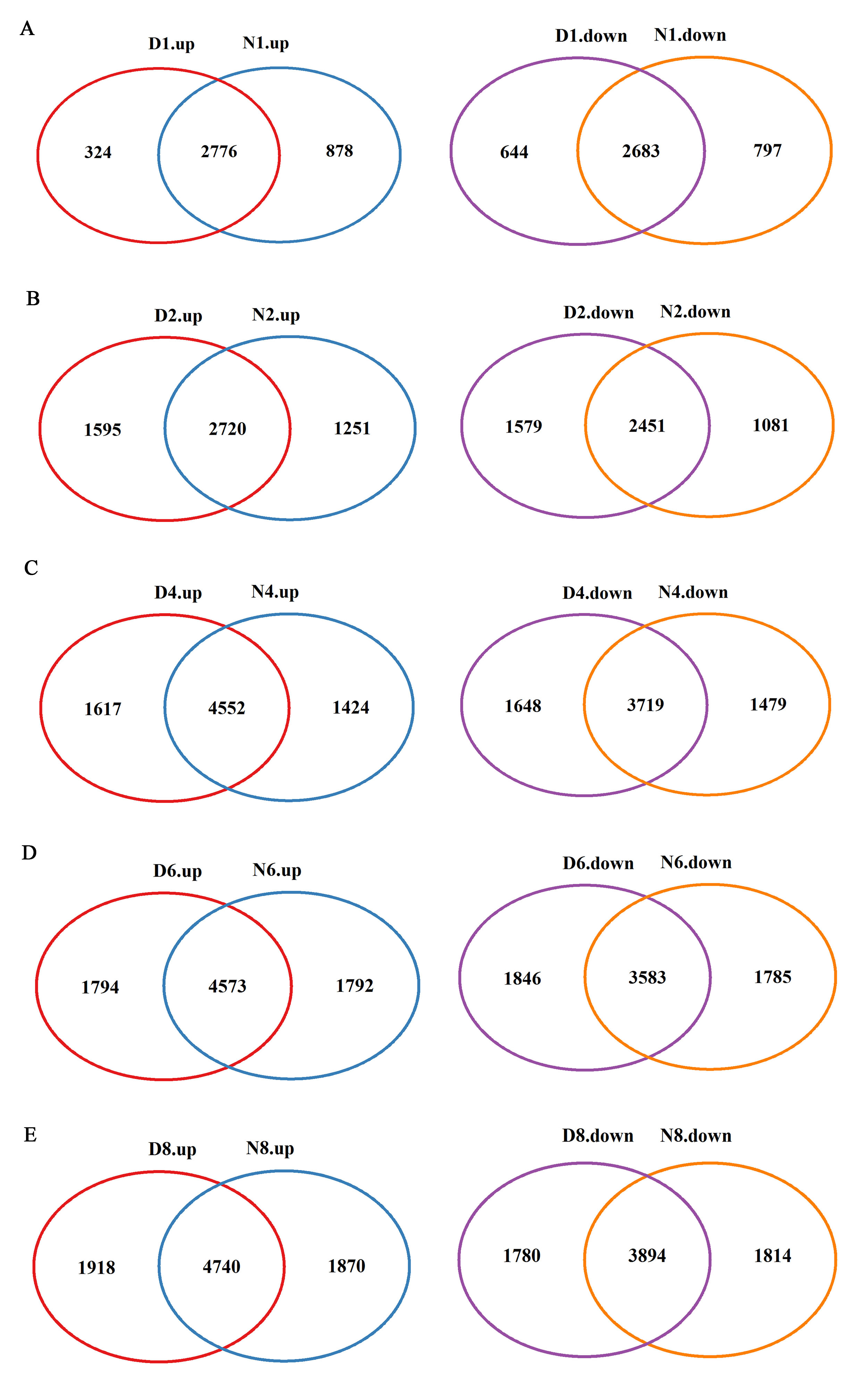

Supplement: Supplementary Figure S1 — Summary of the induced genes among different samples. Comparison of upregulated and downregulated genes at (A) D1, (B) D2, (C) D4, (D) D6 and (E) D8. [file Image_1.jpeg]

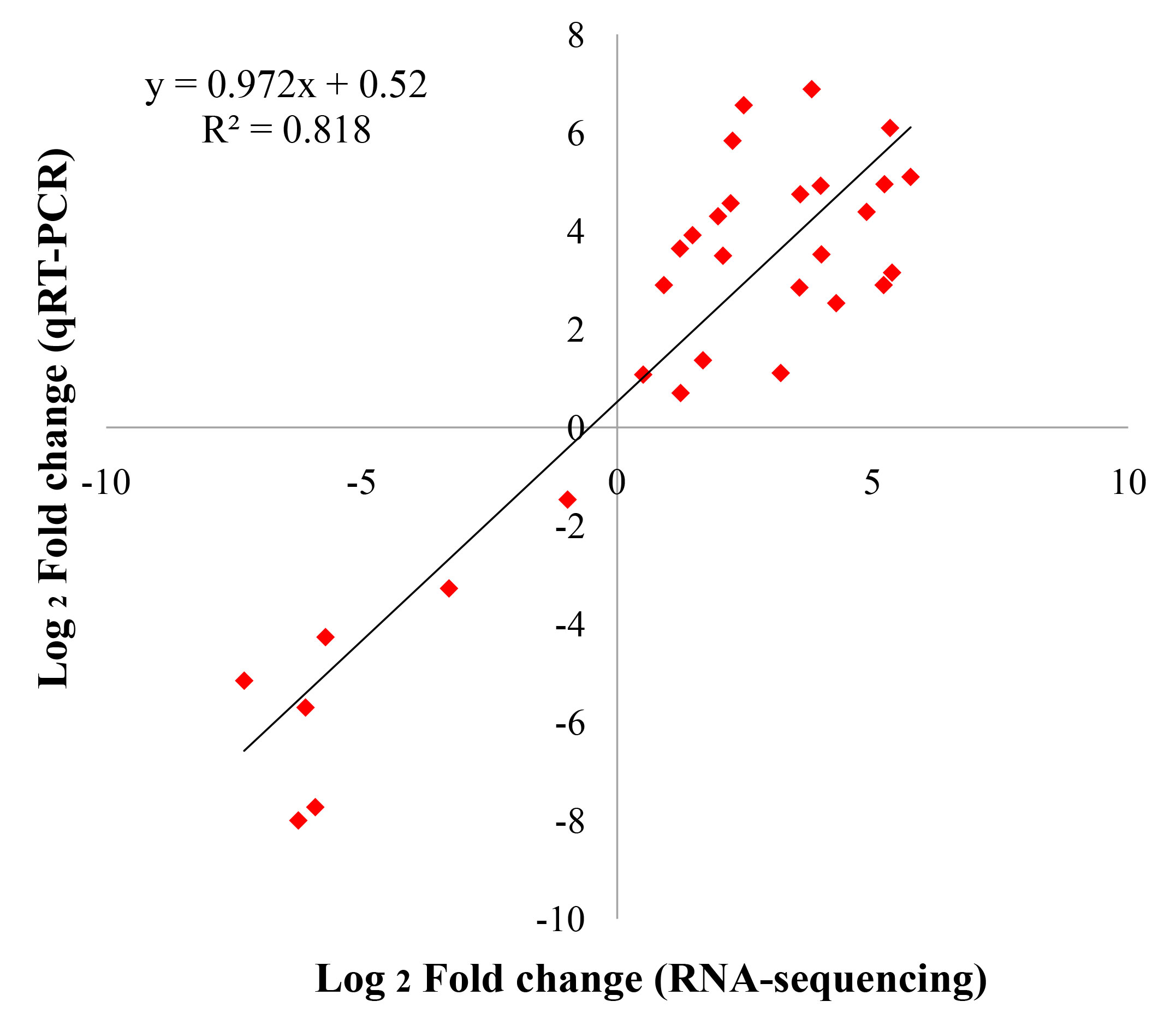

Supplement: Supplementary Figure S2 — Correlations of the differential expression ratios between qRT-PCR and RNA-sequencing of ten induced genes among different samples. [file Image_2.jpeg]

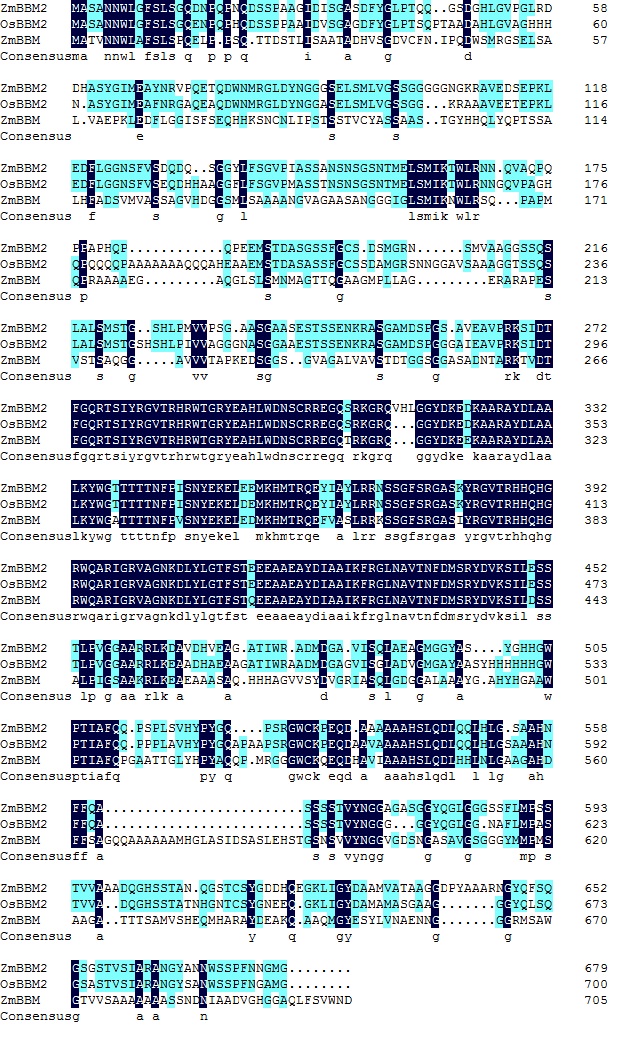

Supplement: Supplementary Figure S3 — Multiple alignment of ZmBBM2 (GRMZM2G366434), OsBBM2 (LOC_Os02g40070) and ZmBBM (GRMZM2G141638) protein sequences. The identical residues are shaded in dark blue and conservative changes are shaded in light blue. [file Image_3.jpeg]

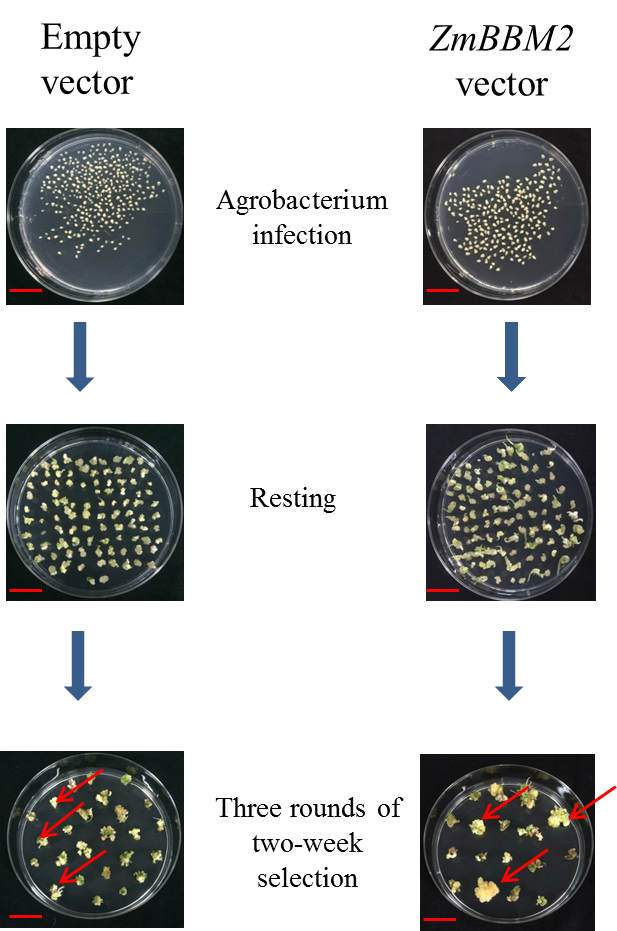

Supplement: Supplementary Figure S4 — Process of the positive callus selection. Immature maize embryos were infected with agrobacterium and co-cultured for three days, and then rested for seven days. The positive calli were selected on medium containing 3 mg/L bialaphos through three rounds of two-week selection. Arrows refer to the positive callus. scale bar=2 cm. [file Image_4.png]

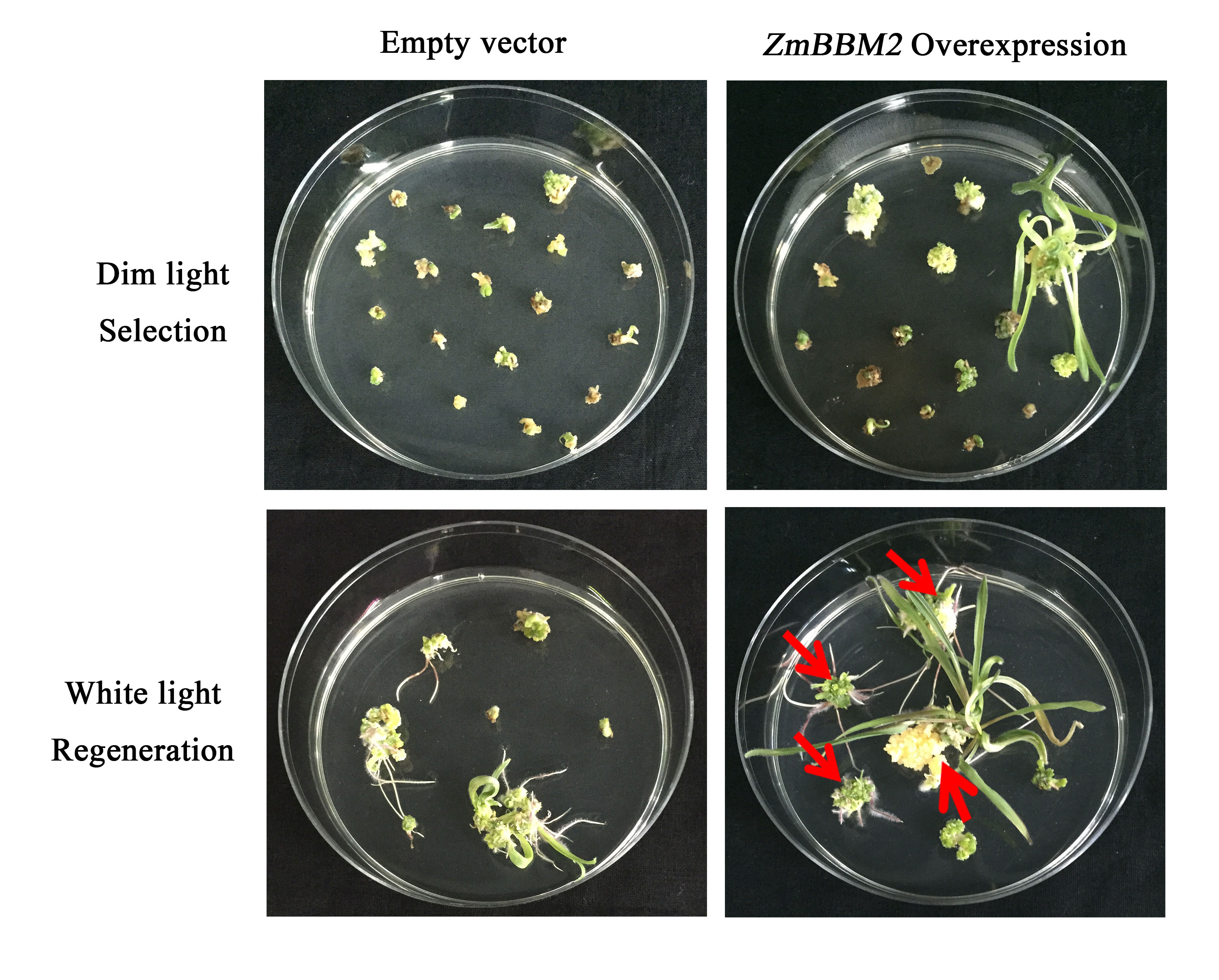

Supplement: Supplementary Figure S5 — Overexpression of ZmBBM2 promotes callus formation and proliferation in the B73. Arrow refers to the ZmBBM2 positive callus. [file Image_5.jpeg]

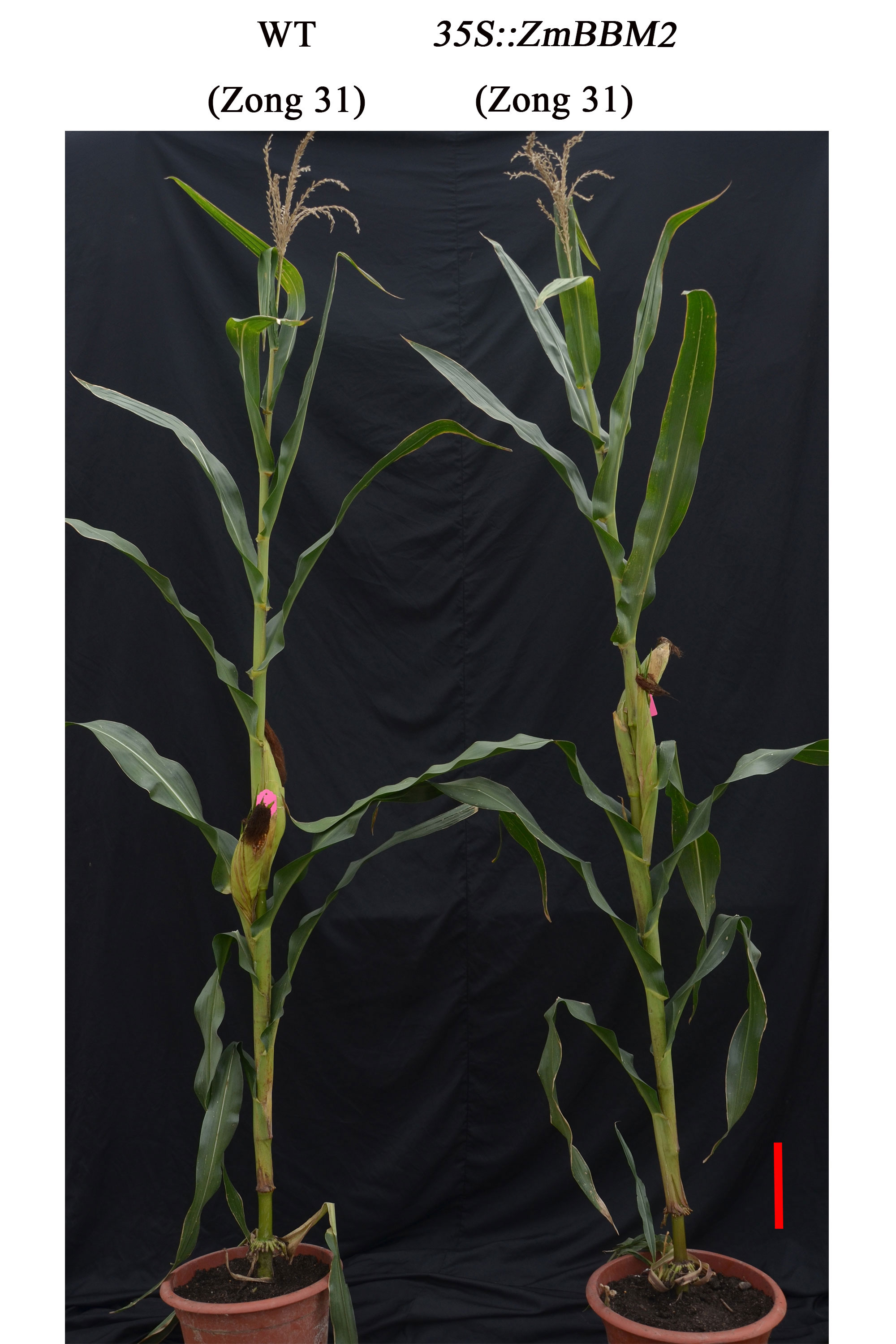

Supplement: Supplementary Figure S6 — The wide type and ZmBBM2 overexpression plants in the Zong31 line at 30 days after self-pollination. scale bar=20 cm. [file Image_6.jpeg]
